# Associations between Screen Time and Physical Activity among Spanish Adolescents

**DOI:** 10.1371/journal.pone.0024453

**Published:** 2011-09-01

**Authors:** Jose A. Serrano-Sanchez, Sara Martí-Trujillo, Angela Lera-Navarro, Cecilia Dorado-García, Juan J. González-Henríquez, Joaquín Sanchís-Moysi

**Affiliations:** 1 Department of Physical Education, University of Las Palmas de Gran Canaria, Campus Universitario de Tafira s/n, Las Palmas de Gran Canaria, Spain; 2 Department of Mathematics, University of Las Palmas de Gran Canaria, Campus Universitario de Tafira s/n, Las Palmas de Gran Canaria, Spain; 3 Department of Physical Education, University of A Coruña, A Coruña, Spain; University of Liverpool, United Kingdom

## Abstract

**Background:**

Excessive time in front of a single or several screens could explain a displacement of physical activity. The present study aimed at determining whether screen-time is associated with a reduced level of moderate to vigorous physical activity (MVPA) in Spanish adolescents living in favorable environmental conditions.

**Methodology/Principal Findings:**

A multi-stage stratified random sampling method was used to select 3503 adolescents (12–18 years old) from the school population of Gran Canaria, Spain. MVPA, screen-time in front of television, computer, video game console and portable console was assessed in the classroom by fulfilling a standardized questionnaire. Bivariate and multivariate logistic regression analyses adjusted by a set of social-environmental variables were carried out. Forty-six percent of girls (95% CI±2.3%) and 26% of boys (95% CI±2.1%) did not meet the MVPA recommendations for adolescents. Major gender differences were observed in the time devoted to vigorous PA, video games and the total time spent on screen-based activities. Boys who reported 4 hours•week^−1^ or more to total screen-time showed a 64% (OR = 0.61, 95% CI, 0.44–0.86) increased risk of failing to achieve the recommended adolescent MVPA level. Participation in organized physical activities and sports competitions were more strongly associated with MVPA than screen-related behaviors.

**Conclusions/Significance:**

No single screen-related behavior explained the reduction of MVPA in adolescents. However, the total time accumulated through several screen-related behaviors was negatively associated with MVPA level in boys. This association could be due to lower availability of time for exercise as the time devoted to sedentary screen-time activities increases. Participation in organized physical activities seems to counteract the negative impact of excessive time in front of screens on physical activity.

## Introduction

Increasing moderate to vigorous physical activity (MVPA) in children and adolescents is one of the major public health strategies advised to reduce obesity and its associated morbidity [1]–[3]. In these populations, MVPA is associated with health benefits, including lower fat accumulation, cardiovascular risk reduction and greater bone mass acquisition [4]–[6]. Despite the slight increase in free time physical activity (PA) in the past decades, sedentary behaviors have also raised potentially blunting the benefits of PA [7], [8]. Sedentary screen-time, especially television (TV)-associated screen-time, is the main contributor to total inactivity in children and adolescents [9]–[12].

It has been suggested that the relationship between screen-time and obesity may be of little clinical significance in children and youth [13], [14]. However, longitudinal studies have shown that screen-time, particularly TV time, increased the risk of being overweight or obese [15]–[18]. Moreover, the excessive TV time has been linked to an increased risk of insulin resistance [19], [20], alterations in lipid profile [21], [22], back pain and headaches [23], and respiratory symptoms [24] in children and adolescents. Since sedentary habits increase during adolescence [25], [26], several countries have established recommendations to limit screen time. In the USA, the American Academy of Pediatrics (AAP) recommends less than 2 hours per day of TV-watching [27]. In Australia and Canada, similar recommendations targeting children and adolescents have been issued [28], [29].

It has been suggested that the relationship between adiposity and cardiovascular risk with screen-time is in part due to the substitution of some PA by sedentary behaviors [30], [31]. In young Finns, Tammelin et al. [32] found that boys and girls who devoted more than 4 hours per day (h•d^−1^) to TV-watching had respectively a 1.4- to 2.5-fold increased risk of not achieving the appropriate level of MVPA compared to a group watching TV for less than 1 h•d^−1^. In contrast, others have postulated that PA and sedentary behaviors should not be considered as opposite ends of the same continuum, as they may be compatible with each other and independent in their relationship to health [33]–[36]. Thus, issuing of recommendations to limit screen-time may not be the best strategy in all countries for increasing adolescent PA levels [37]. In fact, in regions with excellent weather conditions, like Canary Islands, the amount and intensity of physical activities of adolescents may be sufficient to counteract the time expended in front of screens.

Therefore, the aim of the present study was to determine whether screen-time (TV, computer, video games and portable mini-consoles) is associated with a reduced level of MVPA in adolescents living in a region with a climate ideal for physical activity. We hypothesized that adolescents accumulating higher daily screen-times, in total or through a single screen such as TV or computer, would have a greater risk of not achieving the recommended levels of MVPA.

## Materials and Methods

### Sample and data collection

The study took place in Gran Canaria (Spain), which has a stable climate throughout the year, with average annual temperatures between 18–24°C, 21 rainy days per year, and 65–70% environmental humidity levels. A cross-sectional sample of 4000 adolescents (12–18 years-old) from the school population were selected by a multi-stage stratified random sampling method. Sample size was calculated following the procedure described by Bennet et al. [38]. Schools were considered as the basic sample units. A sample error below 2% at a confidence level of 95% was assumed for estimations of the recommended PA. The surveys were stratified proportionally to the adolescents population in local counties and municipalities (20 strata in total). Schools in each stratum were randomly sampled and from each school one or two classrooms were randomly selected for the study. Overall, 127 schools and 227 classrooms with an average of 17.6 pupils per classrooms were interviewed in June 2004. Interviews were carried out in the classroom by trained interviewers using a self-administered questionnaire. The role of interviewers was to present and explain the questionnaire, offering support in the classroom to the participants. The reliability of the questionnaire was tested 2–4 weeks later by repeating the questionnaire in 150 adolescents. After data collection, 12% of surveys were discarded due to lack of PA data or inconsistent responses. The resulting 3,503 surveys provided an overall sampling error of 1.7% at a confidence level of 95.5%. The gender subgroups had a sampling error of 2.4%. The study was performed in accordance with the Helsinki Declaration of 1975, last modified in 2000, as regards the conduct of clinical research. The bioethics committee of the University of Las Palmas de Gran Canaria approved the study. Authorization for the access to school classrooms was previously obtained from the General Director of Education Department. Participants were informed about the objectives of the survey and verbal consent was obtained from them, and written consent from their parents. Data were analyzed anonymously.

### Measurements

MVPA was assessed with the Minnesota Leisure Time Physical Activity Questionnaire (MLTPAQ), and the assessment period was limited to activities in the month preceding the interview. The questionnaire registered the number of days and the average duration of 58 recreational and occupational MVPAs lasting a minimum of 10 minutes. The physical activities of the MLTPAQ are classified according to their level of intensity expressed in metabolic equivalents (MET-min.), obtained from the Compendium of Physical Activities [39], [40]. The time and energy spent in moderate PA (3.5–5.9 MET-min), vigorous PA (≥6 MET-min) and the total PA (≥3.5 MET-min) were calculated from the responses given in the questionnaire. The MLTPAQ has been validated in adolescents against doubly labeled water (r = 0.49 and r = 0.74, when sedentary activities such as time in front of screens and sleep were included) [41]. To determine associations between MVPA and screen-time, we used a dichotomous variable that classified the adolescents into two MVPA categories using the recommendation of 60 minutes of daily activity as the cutoff point [42]–[44]. Adolescents were classified as sufficiently active if they met the following three conditions: 1) at least 5 days per week (d•w^−1^) of MVPA (≥3.5 MET), 2) at least 300 minutes per week (min•w^−1^) of MPVA and 3) at least 1,800 MET-min•s^−1^ (300 min•w^−1^ •6 MET-min medium-intensity) of MPVA; otherwise participants were classified as insufficiently active.

Sedentary screen-time was measured with the specific items on the Television and Video Measures (TVM) questionnaire [45]. The TVM records the daily hours and minutes spent on TV, computer and video games in a typical week. The screen items have been validated in American children (aged 11–13 years) and Mexican children (aged 10–14 years) with correlation coefficients of 0.54 and 0.53, respectively, using a criterion of 24-hour recall [45], [46]. In our study, we specifically asked for the number of days a week and the average time per day spent on TVs, computers, video games and portable mini-consoles in weekdays and weekends. This procedure has been tested for reliability in young Australians (aged 11–15 years) showing correlation coefficients between 0.72 and 0.90 [47]. We used the total hours per weak (h•w^−1^) as the output variable for behaviors in front of screens. Intraclass correlation coefficients (ICC) between the first and second measurements for the logarithm of the number of h•w^−1^ for each of the behaviors in front of screens were ICC_TV_ = 0.86, ICC_computer_ = 0.81, ICC_videogames_ = 0.88, ICC_mini-consoles_  = 0.90 and ICC_total_ = 0.84. The relative importance of screen-time compared to other social environmental factors associated with MVPA [48], [49] was assessed. The following characteristics were surveyed and coded: 1) gender (1. boy and 2. girl), 2) age (continuous, 12 to 18 years), 3) PA of the father (1. regularly [≥3 d•w^−1^ for 30 minutes or more], 2. sporadically [≥1 d•w^−1^ for 10 minutes or more] and 3. inactive (<1 d•w^−1^), 4) PA of the mother (same criteria than for the father), 5) organized participation (1. organized [directed by an instructor] and 2. not organized), 6) participation in sports competitions in the previous year (1. at least one and 2. none), 7) appeal of the physical education classes (1. I do not like them at all, 2. I do not like them, 3. indifferent, 4. I like them and 5. I like them a lot; coded into three groups: 1–2–3. low appeal, 4. medium appeal and 5. high appeal) and 8) access to PA spaces (1. indoor 2. outdoor 3. both and 4. none).

### Statistical analysis

Statistical analysis were conducted separately for boys and girls. Since MVPA time and time engaged in screen-based behaviors (h•w^−1^) did not follow a normal distribution percentiles and medians were calculated. The 95% confidence interval (95% CI) of the median was calculated using the Daly and Bourke formula [50]. Differences between boys and girls in the prevalence of the recommended levels of screen-time and MVPA were tested with chi-square test. Associations between MVPA and screen-based behaviors were analyzed using unconditional multiple logistic regression, with the activity level of the subjects as the binary dependent variable (sufficiently versus insufficiently active). The logistic regressions were unadjusted (bivariate) and adjusted for all screen-based behaviors in addition to the social environmental variables listed above, including age and municipality size. To assess the assumption of a linear relationship, two types of logistic regression analyses were performed: one, using a continuous variable of screen-time (h•d^−1^) and the other, classifying the participants into 3 categories according to screen-time (≤2 h•d^−1^, >2 h•d^−1^ and ≥4 h•d^−1^). The ≤2 h•d^−1^ category was used as the reference category. The odds ratio, the associated 95% CI and the goodness of fit with the Hosmer and Lemeshow test (HL test) were determined. Statistical analysis was performed with the SPSS v 15.0 software [51]. Statistical significance was assumed when p<0.05.

## Results

### Sample

The final sample was compared with previously collected Spanish census data (Table 1). Age, gender and grade groups presented differences ranging from 0.5 to 4.8% compared to the national census data (2008). The data for all socio-demographic groups, with the exception of one, had a sampling error for the 95% CI that was within±2.4–3.5%.

**Table 1:** Characteristics of age, gender and municipality size of study participants.

	Sample			National Census
	N	%	% error	%
**Gender**				
Male	1 695	48.4	±2.4	51.4
Female	1 808	51.6	±2.4	48.6
**Age**				
12 - <14	920	26.3	±3.3	26.8
14 - <16	1173	33.5	±2.9	28.7
16–18	1410	40.3	±2.7	44.4
**Municipality size**				
≤20,000 inhab.	1189	34.0	±2.9	34.9
20,001–50,000 inhab.	823	23.5	±3.5	15.4
50,001–100,000 inhab.	374	10.7	±5.2	11.3
≥100,001 inhab.	1117	31.9	±3.0	38.4

### Physical activity levels

Table 2 reports the levels of the MVPA and the main PA of the adolescent participants. In total, 26.2% of boys (95% CI, 24.1–28.3%) and 46% of girls (95% CI, 43.7–48.3%) did not reach the recommended levels of MVPA. Walking (10 minutes or more) was the most prevalent PA in girls (80.3%, 95% CI, 78.5–82.1%). In boys, the most prevalent MVPA was playing soccer (66.2%, 95% CI, 63.9–68.5%), followed by walking (57.5%, 95% CI, 55.1–59.9%). Thirty seven percent of subjects participated in fitness activities in gyms in the previous month. Compared to girls, boys had a 2.5 to 12.7-fold higher MVPA participation in biking, outdoor activities, weight-lifting training, soccer, motor sports and combat sports (p <0.05). Girls had a 1.4 to 28-fold higher MVPA participation in walking, dancing, roller sports and aerobics than boys (p<0.05). There were no between-sex differences or the differences were small in jogging, swimming, treadmill and racquet sports (excluding table tennis).

**Table 2:** Prevalence of physical activities and screen-related behaviors among adolescent participants in Gran Canaria Physical Activity Study.

	Overall			Boys			Girls		
	n	%	95% CI	n	%	95% CI	n	%	95% CI
**Total of screen-time** *									
≤2 h/day	1138	33.4	±1.6	488	29.5	±2.2	650	37.1	±2.3
>2<4 h/day	1085	31.9	±1.6	532	32.2	±2.3	553	31.6	±2.2
≥4 h/day	1180	34.7	±1.6	633	38.3	±2.3	547	31.3	±2.2
**Television** *									
≤2 h/day	2099	61.6	±1.6	1093	65.9	±2.3	1006	57.5	±2.3
>2 h/day	1311	38.4	±1.6	566	34.1	±2.3	745	42.5	±2.3
**Computer**									
≤2 h/day	2960	84.9	±1.2	1425	84.7	±1.7	1535	85.0	±1.6
>2 h/day	527	15.1	±1.2	257	15.3	±1.7	270	15.0	±1.6
**Video-games** *									
≤2 h/day	3350	96.0	±0.7	1565	92.9	±1.2	1785	98.9	±0.5
>2 h/day	140	4.0	±0.7	120	7.1	±1.2	20	1.1	±0.5
**Portable mini-console** *									
≤2 h/day	3463	99.2	±0.3	1663	98.5	±0.6	1754	99.8	±0.2
>2 h/day	29	0.8	±0.3	25	1.5	±0.6	4	0.2	±0.2
**Physical activity level** * a									
Sufficiently active	2228	63.6	±1.6	1251	73.8	±2.1	977	54.0	±2.3
Insufficiently active	1275	36.4	±1.6	444	26.2	±2.1	831	46.0	±2.3
**Physical activities**									
** Walking**	2426	69.3	±1.5	975	57.5	±2.4	1451	80.3	±1.8
Occupational	1787	51.0	±1.7	686	40.5	±2.3	1101	60.9	±2.2
Recreational	1373	39.2	±1.6	547	32.3	±2.2	826	45.7	±2.3
** Fitness**	1324	37.8	±1.6	491	29.0	±2.1	833	46.1	±2.3
Aerobics b	631	18.0	±1.3	21	1.2	±0.5	610	33.7	±2.2
Static cycling, treadmill	464	13.2	±1.2	221	13.0	±1.6	243	14.0	±1.6
Weight lifting	416	11.9	±1.1	298	17.6	±1.8	118	6.5	±1.1
** Soccer**	1429	40.8	±1.6	1122	66.2	±2.3	307	17.0	±1.7
** Others team sports** c	1199	34.2	±1.6	664	39.2	±2.3	535	29.6	±2.1
** Dancing**	1130	32.3	±1.5	313	18.5	±1.8	817	45.2	±2.3
** Footing, jogging**	845	24.1	±1.4	403	23.8	±2.0	442	24.4	±2.0
** Racquet sports** d	638	18.2	±1.3	405	23.9	±2.0	233	12.9	±1.5
Excluding table tennis	343	9.8	±1.0	178	10.5	±1.4	165	9.1	±1.3
** Swimming**	600	17.1	±1.2	264	15.6	±1.7	336	18.6	±1.8
** PA in natural environments** e	543	15.5	±1.2	453	26.7	±2.1	90	5.0	±1.0
** Cycling**	371	10.6	±1.0	297	17.5	±1.8	74	4.1	±0.9
Ocupational	277	7.9	±0.9	221	13.0	±1.6	56	3.1	±0.8
Recreational	161	4.6	±0.7	131	7.7	±1.3	30	1.7	±0.6
** Roller sports and PA** f	307	8.8	±0.9	48	2.8	±0.8	259	14.3	±1.6
** Combat sports** g	181	5.2	±0.7	127	7.5	±1.3	54	3.0	±0.8
** Motor sports** h	140	4.0	±0.6	129	7.6	±1.3	11	0.6	±0.4

a 5 days per week of AFMV +300 minutes per week +1,800 MET per week;

b Aerobics, step and similar;

c Basketball, handball, volleyball, rugby, field hockey, baseball;

d Tennis, badminton, paddle, squash, table tennis;

e Surfing, windsurfing, diving, hunting, canoeing, rowing, sailing;

f Skateboard, skate;

g Judo, karate, taekwondo, fencing, wrestling;

h Motocross, trial, motorcycling in track, karts;

* p<0.05 for differences between boys and girls.

### Physical activity and screen-time

**Figure 1:**
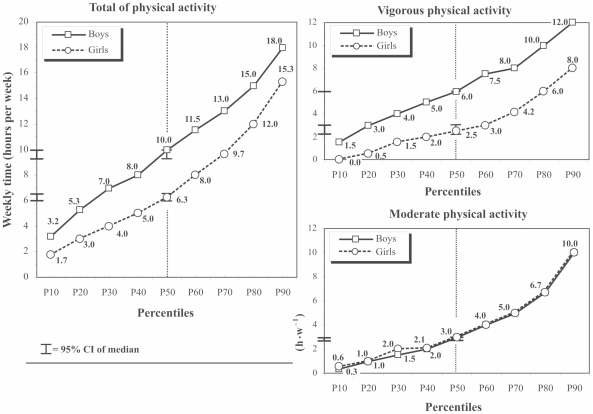
Percentiles of time dedicated to moderate, vigorous and total of physical activities among adolescents participants.

The boys achieved a higher median of total MVPA time (3.7 h•w^−1^ more than girls, p<0.05) (Figure 1) due to the higher levels of vigorous PA of boys (p<0.05). The median of vigorous PA time was 6 h•w^−1^ (95% CI 6.0–6.0) and 2.5 h•w^−1^ (95% CI, 2.3–3.0), in boys and girls, respectively (p<0.05). Both sex showed almost identical levels of moderate PA time (Figure 1).

**Figure 2:**
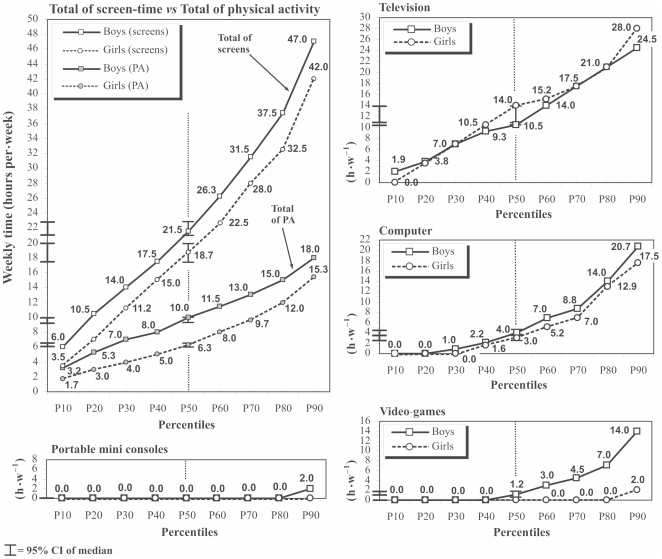
Percentiles of time dedicated to television, computer, video-game, portable mini consoles and total of screen-related behaviors among adolescents participants.

The TV screen-time median was 10.5 h•w^−1^ (95% CI, 10.5–10.5) and 14 h•w^–1^ (95% CI, 10.5–14.0) in boys and girls, respectively (Figure 2). In total, 34% boys (95% CI, 32–36%) and 43% of girls (95% CI, 40–45%) were above the recommended time for TV-watching (>2 h•d^−1^ or >14 h•w^−1^) (p<0.05) (Table 2). Computer use was the second most prevalent screen-based behavior among adolescents and reached a similar level in boys and girls (3–4 h•w^−1^). Fifteen percent of boys (95% CI, 14–17%) and girls (95% CI, 13–17%) were above 2 h•d^−1^ of computer use (Table 2). Although for boys the weekly median video game playing time was 1.2 h•w^−1^ (95% CI, 1.0–1.8), approximately 40% of them had not played any video games in the previous month (Figure 2), and 7% reported more than 2 h.d^−1^. For girls, video game playing was uncommon; 1.1% of girls (95% CI, 0.6–1.6%) reported more than 2 h•d^−1^ of video game playing. The interest in the use of portable mini-consoles was below 15% in boys and girls (12–18 years-old).

Boys devoted about 3 h•w^−1^ more time than girls to total screen time (p <0.05). In total, 38% of boys (95% CI, 36–40%) and 31% of girls (95% CI, 29–33%) reported spending ≥4 h•d^−1^ (28 h•w^−1^) in front of screens (p<0.05). The time spent watching TV represented 55% (95% CI, 53–56%) and 66% (95% CI, 65–68%) of the total screen time in boys and girls, respectively (p<0.05). Boys spent 2.2-fold more time in front of screens than in MVPA (95% CI, 2.1–2.4), while the corresponding value for girls was 3.0-fold (95% CI, 2.7–3.2).

### Associations between screen-time and physical activity

Logistic regression results are presented in Table 3. Results of bivariate analysis showed that boys who achieved ≥4 h•d^−1^ in either TV watching, computer use, video game playing or total screen time had significantly increased probability of being not sufficiently active. In girls, there were no significant bivariate associations between screen-related behaviors and achievement of the recommended levels of MVPA. In boys, after adjustment, all screen-time-associated variables lost their association when analyzed separately, except total screen-time. For each one hour increase in total screen-time, the MVPA level fell by 7.5% (OR = 0.93, 95% CI, 0.88–0.99) in boys. The group which had a total screen-time ≥4 h•d^−1^ was the main contributor to this association, showing a 64% risk (OR = 0.61, 95% CI, 0.44–0.86) of failing to meet the recommended level of MVPA. The HL goodness-of-fit-test for the adjusted model with categorical variables of screen-time achieved values of 0.44 and 0.21 in boys and girls, respectively. For the adjusted model with the continuous variable of total screen-time, the HL was 0.58 and 0.32 in boys and girls, respectively. The percentage of correctly predicted cases was between 65–77%.

**Table 3:** Odds ratio for being sufficiently active in relation to screen-related behaviors and social environments variables among adolescents participants in Gran Canaria Physical Activity Study.

	Unadjusted								Adjusted							
	Boys				Girls				Boys				Girls			
	OR	95% CI			OR	95% CI			OR	95% IC			OR	95% IC		
**Total of screen-time** a	0.95	(0.91,	0.99)	*	1.00	(0.96,	1.05)		0.93	(0.88,	0.99)	*	1.03	(0.98,	1.08)	
≤2 h/day	1.00	(ref.)			1.00	(ref.)			1.00	(ref.)			1.00	(ref.)		
>2 <4 h/day	0.84	(0.63,	1.12)		1.05	(0.83,	1.32)		0.76	(0.58,	0.99)	*	1.06	(0.81,	1.40)	
≥4 h/day	0.76	(0.58,	0.99)	*	1.00	(0.79,	1.25)		0.61	(0.44,	0.86)	*	1.13	(0.85,	1.49)	
**Television** a	0.90	(0.83,	0.98)	*	0.96	(0.90,	1.03)		0.91	(0.83,	1.01)		1.01	(0.94,	1.10)	
≤2 h/day	1.00	(ref.)			1.00	(ref.)			1.00	(ref.)			1.00	(ref.)		
≥4 h/day	0.60	(0.42,	0.88)	*	0.77	(0.58,	1.03)		0.66	(0.41,	1.06)		1.09	(0.75,	1.58)	
**Computer** a	0.94	(0.86,	1.02)		1.06	(0.98,	1.14)		0.90	(0.81,	1.00)		1.02	(0.93,	1.12)	
≤2 h/day	1.00	(ref.)			1.00	(ref.)			1.00	(ref.)			1.00	(ref.)		
≥4 h/day	0.55	(0.35,	0.87)	*	1.42	(0.83,	2.44)		0.56	(0.31,	1.01)		1.18	(0.61,	2.29)	
**Video-game** a	1.00	(0.89,	1.12)		1.21	(0.97,	1.51)		1.08	(0.93,	1.26)		1.11	(0.97,	1.37)	
≤2 h/day	1.00	(ref.)			1.00	(ref.)			1.00	(ref.)			1.00	(ref.)		
≥4 h/day	0.47	(0.23,	0.97)	*	1.70	(0.15,	18.8)		1.11	(0.36,	3.39)		0.86	(0.05,	14.9)	
**Portable mini-console** a	0.89	(0.69,	1.15)		0.90	(0.60,	1.34)		0.76	(0.55,	1.05)		0.66	(0.39,	1.11)	
≤2 h/day	1.00	(ref.)			1.00	(ref.)			1.00	(ref.)			1.00	(ref.)		
>2 a <4 h/day	0.86	(0.36,	2.10)		0.28	(0.03,	2.72)		0.60	(0.16,	2.21)		0.14	(0.01,	1.63)	
**PA of father**																
Inactive	1.00	(ref.)			1.00	(ref.)			1.00	(ref.)			1.00	(ref.)		
Regular (≥3 d/w^-1^)	1.76	(1.34,	2.31)	*	1.74	(1.38,	2.19)	*	1.47	(1.03,	2.11)	*	1.23	(0.92,	1.65)	
**PA of mother**																
Inactive	1.00	(ref.)			1.00	(ref.)			1.00	(ref.)			1.00	(ref.)		
Regular (≥3 d/w^-1^)	1.52	(1.16,	1.98)	*	1.71	(1.37,	2.14)	*	1.13	(0.79,	1.61)		1.36	(1.02,	1.82)	*
**Organized participation**																
Non organized	1.00	(ref.)			1.00	(ref.)			1.00	(ref.)			1.00	(ref.)		
Organized	2.93	(2.33,	3.68)	*	2.90	(2.39,	3.52)	*	2.07	(1.54,	2.78)	*	2.12	(1.66,	2.71)	*
**Sports competitions**																
None	1.00	(ref.)			1.00	(ref.)			1.00	(ref.)			1.00	(ref.)		
At least one (last year)	3.15	(2.51,	3.97)	*	2.66	(2.07,	3.42)	*	2.42	(1.81,	3.24)	*	1.83	(1.33,	2.52)	*
**Appeal of PE classes** b	1.42	(1.25,	1.61)	*	1.42	(1.28,	1.57)	*	1.25	(1.07,	1.47)	*	1.32	(1.16,	1.49)	*
Low	1.00	(ref.)			1.00	(ref.)			1.00	(ref.)			1.00	(ref.)		
Medium	1.14	(0.76,	1.70)		1.32	(0.99,	1.76)		0.90	(0.54,	1.49)		1.21	(0.86,	1.72)	
High	2.14	(1.51,	3.03)	*	2.43	(1.85,	3.20)	*	1.52	(1.07,	2.37)	*	1.97	(1.40,	2.77)	*
**Access to PA spaces**																
None	1.00	(ref.)			1.00	(ref.)			1.00	(ref.)			1.00	(ref.)		
Outdoor	1.25	(0.79,	1.97)		1.58	(1.08,	2.31)	*	0.98	(0.55,	1.77)		1.36	(0.83,	2.20)	
Indoor	1.25	(0.69,	2.24)		1.54	(1.02,	2.33)	*	1.04	(0.50,	2.20)		1.22	(0.73,	2.05)	
Outdoor and Indoor	1.83	(1.18,	2.84)	*	2.62	(1.86,	3.68)	*	1.42	(0.81,	2.50)		1.85	(1.20,	2.86)	*

a For each increase of 1 hour per day;

b For each increase of 1 point in scale (0 =  I don’t like it at all, 5 =  I like it a lot);

*p<0.05.

Social environmental variables were more strongly associated with MVPA than total screen-time. Participation in sports competitions and organized participation (physical activities directed by an instructor) demonstrated the most consistent association with achievement of the recommended MVPA in boys (Table 3). The appeal of physical education classes was positively associated with MVPA in boys and girls who answered that they liked the classes a lot compared to the adolescents who said they felt indifferent or low-attracted for the physical education classes. For each increase of 1 point (in a scale going from 1 to 5, being 5 the maximum level of appeal) the likelihood of achieving a sufficient level of MVPA increased by 25% in boys (p <0.05) and 32% in girls (p <0.05). Regular PA (≥3 d•w^−1^) of the father in case of boys and the mother in case of girls was positively associated with the likelihood of being sufficiently active (Table 3). The perceived access to PA spaces to a combination of outdoor and indoor facilities was positively associated with the likelihood of being sufficiently active in girls.

To assess the relative contribution of each of the variables in the final variance explained by the multivariate analysis, we calculated the change (percentage) in the coefficient of determination (Nagelkerke's pseudo R^2^) [52] using a step by step analysis. The variables that explained most of the variance of the multivariate analysis (pseudo R^2^) for the boys were participation in competitions (33%) and organized participation (20%). The corresponding variables in girls were organized participation (30%) and the appeal of the PE school courses (27%). Additionally, we checked the effect of removing organized participation and participation in competitions from the multivariate model. We noted that the associations between each sedentary behavior and MVPA levels tended to recover their value before adjustment in boys (Table 2). We also performed a series of logistic regression analyses using the recommended time for each screen-based activity (≤2 h•d^−1^) as dependent variables. Results showed that participation in organized PA decreased the risk of excessive use of TV by 33% in both sexes and video games by 59% in boys (all p<0.05).

## Discussion

In agreement with our hypothesis, the total time spent in front of screens was associated with reduced physical activity in adolescents. However, this was not the case when each type of screen-related activity was analyzed separately, after adjustment for social environmental variables. The latter implies that the association between TV-watching and obesity should be explained by additional mechanisms apart from displacement of PA. For example, increased energy intake while watching TV associated with advertising persuasion to consume energy-rich foods [53]–[55]. In agreement, a small reduction in BMI, skinfold thickness and waist circumference was observed in an intervention study aiming at reducing the screen-time in children, even though no statistically significant differences in MVPA were observed between the intervention and the control group [56].

Our findings also concur with the study by Feldman et al. [57], who found no association between PA and the amount of time spent in sedentary screen-based activities in young Canadian high school students. Studies conducted with Portuguese and Turkish children and adolescents showed that screen-time and TV viewing did not discriminate between the active and inactive adolescents [58], [59]. Similarly, a study with European adolescents found no associations between MVPA (measured with accelerometers) and the amount of time spent watching TV (assessed by questionnaire), suggesting that these two factors independently influence health [60]. It has been reported that inactivity is associated with several sedentary behaviors apart from TV-watching [37]. Our study support that the excess time accumulated in front of screens is negatively associated with MVPA. Consequently, making recommendations to limit the time in front a single screen (i.e., TV) may not be effective in increasing MVPA [35], since adolescents may replace the screen time by other sedentary behaviors.

Paradoxically, computer-use time has been positively associated with PA [37], [57], [59]. In the present investigation computer use was only associated with reduced PA in the group with more than 4 h.d^−1^ of computer use. However, this association was not significant after adjustment.

Differences in the associations between MVPA and screen-based behaviors depending on the type of analysis (each behavior separately or all screen-based behaviors together) may be due to the time management of adolescents and children. Hager et al. [61] reported a negative association in 9- to 12-year-old children between screen-time and PA (measured with accelerometers) after school time periods but not in other moments, suggesting a displacement of PA only at certain times of day. This is consistent with the organization and management of time by children and adolescents according to mandatory, occupational and recreational activities depending on the different times of the day [62]. Thus, an increase of video game time, for example, would not necessarily affect occupational MVPAs. In our study, occupational activities, primarily walking, contributed markedly to the total MVPA. This could explain the lack of association between MVPA and individual screen-based sedentary activities in the present investigation. However, if the accumulation of time in screen-based activities occurs during different periods of day, a reduction of MVPA is more likely.

In the present study, unadjusted screen-related behaviors were associated with MVPA among boys (as this was not the case among girls). However, these associations disappeared after accounting for the participation in organized PA, which was the principal contributor to the variance of MVPA. Participation in sport competitions in boys, and the appeal of PE classes in girls, were also associated independently to MVPA. These variables accounted for more than 50% of the value of the pseudo-R^2^. By removing these variables from the model, the association between excessive TV-watching (≥4 h•d^−1^), computer time, or playing video games with insufficient MVPA was statistically significant again. Our results showed that the likelihood of achieving a sufficient level of MVPA was twice as high for boys and girls participating in organized PA, regardless of participation in sports competitions. In agreement, children and adolescents who participated in organized activities had a higher level of PA than those in unorganized PA [63]–[65]. Participation in organized sports has also been associated with reduced adiposity, improved lipid profile and enhanced cardio-respiratory fitness [5], [66], [67], as well as better performance in fundamental movement skills when compared to participation in unorganized PA [68].

Our study indicates that participation in organized MVPA and in sports competitions diminishes the risk of an insufficient level of MVPA, even in adolescents spending excessive time in front of TV or video-games. In agreement, Vicente-Rodríguez et al. [69] reported that excessive use of TV (≥3 h•d^−1^) lost its association with decreased bone mass in adolescents who participated in organized sports.

The present study underscores the importance of attractive PE courses, as previously reported [65], [70]–[73]. Some activities associated with a masculine stereotype, such as the recommended use of weights to improve muscle strength and health [1], [74], was hardly achieved by girls from this cohort.

In agreement with other studies, approximately 2/3 of our adolescents followed the recommendations for TV-watching [32], [37], [75], [76]. The 38 and 31% of boys and girls exceeding 4 h•d^−1^ of total screen time had 64% higher risk of failing to achieve the recommended level of MVPA in the present study. Recommendations to limit the total time spent in front of screens [77] are supported by the present study. For cardiovascular risk prevention, the recommendations should account for the total time spent in sedentary activities, including also non-screen-based sedentary activities [34].

### Limitations and strengths

This study has some limitations. The cross-sectional design impedes achievement of causal conclusions. MVPA and sedentary behaviors were assessed with questionnaires, which have lower validity and reliability than objective measures. Despite the limitations, the associations found between MVPA and environmental variables were consistent. The study population is only representative of the Island of Gran Canaria, which is culturally and ethnically comparable to the rest of the Spanish population. The sample was not stratified by the public or private adscription of the schools. Nevertheless, all schools had the same selection possibilities and the sample of schools studied is representative of the schools present in Gran Canaria. As strengths, the evaluation of the involvement in sedentary screen-based activities was based on questionnaires, which have the advantage of analyzing separately the different screen-related behaviors (e.g., TV, computer use). In addition, our study includes a large number of participants and is the first study, to our knowledge, to evaluate the association between sedentary screen-based activity and MVPA among Spanish adolescents.

### Conclusions

Adolescents from Gran Canaria, despite the excellent all year around weather conditions, expend seated in front of screens as much time as that reported in other European countries. The girls were the main group at risk of physical inactivity, representing almost two-thirds of the inactive group. Part of the daily physical activity appears to be replaced by total screen time. Participation in organized physical activities and sports competitions seems to counteract the negative impact of excessive time in front of screens on physical activity. Therefore, it may be advisable to implement policies aiming at increasing the participation in organized physical activities, like sports and competitions.
